# Endogenous Lithium Status in Major Depression: A Retrospective Analysis of EDTA Blood Samples

**DOI:** 10.1192/j.eurpsy.2026.11431

**Published:** 2026-07-31

**Authors:** J. Eder, M. S. Simon

**Affiliations:** Department of Psychiatry and Psychotherapy, LMU University Hospital, Munich, Germany

## Abstract

**Introduction:**

Lithium is a naturally occurring trace element present in rocks and drinking water. Ecological studies suggest that higher environmental lithium exposure is associated with lower suicide rates, reduced psychiatric hospitalizations, and decreased dementia incidence. At the molecular level, lithium modulates neurotrophic and inflammatory pathways, including upregulation of brain-derived neurotrophic factor (BDNF) and inhibition of glycogen synthase kinase-3β (GSK3β), supporting neuroprotection. Physiological lithium may also influence vitamin B12 transport and overall mental health. However, no studies have directly compared endogenous lithium concentrations between individuals with and without major depression.

**Objectives:**

This study aims to determine whether patients with major depressive disorder exhibit lower endogenous lithium levels than matched non-depressed individuals. We hypothesize that reduced physiological lithium may contribute to the pathophysiology of depression and could inform future preventive or microdose therapeutic strategies.

**Methods:**

This project aims to investigate whether depressive patients exhibit lower endogenous lithium levels than matched non-depressed individuals. We will conduct a retrospective, naturalistic secondary analysis of EDTA blood samples from patients with major depressive episodes (F32/F33, SCID-diagnosed) and propensity score-matched controls from primary care cohorts without a psychiatric diagnosis. Patients currently receiving lithium medication will be excluded. Lithium concentrations will be quantified using inductively coupled plasma mass spectrometry (ICP-MS). Statistical analyses will include group comparisons, regression models, and sensitivity analyses.

**Results:**

As this is a planned retrospective study, results are not yet available. We anticipate that depressed patients may exhibit lower endogenous lithium levels than controls. If confirmed, this finding would support a potential biological role for physiological lithium in mood regulation and mental health.

**Conclusions:**

This study will provide the first systematic evaluation of endogenous lithium concentrations in depression. Demonstrating reduced lithium in depressive patients could substantiate the hypothesis that trace lithium contributes to mood regulation and highlight the potential of microdose lithium interventions or preventive strategies. All data and analysis scripts will be shared in accordance with Open Science principles, including preregistration on OSF and publication of anonymized datasets and code.

**Disclosure of Interest:**

None Declared

